# Spike Timing Neural Model of Motion Perception and Decision Making

**DOI:** 10.3389/fncom.2019.00020

**Published:** 2019-04-05

**Authors:** Petia D. Koprinkova-Hristova, Nadejda Bocheva, Simona Nedelcheva, Mirsolava Stefanova

**Affiliations:** ^1^Institute of Information and Communication Technologies, Bulgarian Academy of Sciences, Sofia, Bulgaria; ^2^Institute of Neurobiology, Bulgarian Academy of Sciences, Sofia, Bulgaria

**Keywords:** visual perception, self-motion, spike timing neuron model, visual cortex, LGN, MT, MST, LIP

## Abstract

The paper presents a hierarchical spike timing neural network model developed in NEST simulator aimed to reproduce human decision making in simplified simulated visual navigation tasks. It includes multiple layers starting from retina photoreceptors and retinal ganglion cells (RGC) via thalamic relay including lateral geniculate nucleus (LGN), thalamic reticular nucleus (TRN), and interneurons (IN) mediating connections to the higher brain areas—visual cortex (V1), middle temporal (MT), and medial superior temporal (MTS) areas, involved in dorsal pathway processing of spatial and dynamic visual information. The last layer—lateral intraparietal cortex (LIP)—is responsible for decision making and organization of the subsequent motor response (saccade generation). We simulated two possible decision options having LIP layer with two sub-regions with mutual inhibitory connections whose increased firing rate corresponds to the perceptual decision about motor response—left or right saccade. Each stage of the model was tested by appropriately chosen stimuli corresponding to its selectivity to specific stimulus characteristics (orientation for V1, direction for MT, and expansion/contraction movement templates for MST, respectively). The overall model performance was tested with stimuli simulating optic flow patterns of forward self-motion on a linear trajectory to the left or to the right from straight ahead with a gaze in the direction of heading.

## Introduction

Vision has to encode and interpret in real time the complex, ambiguous, and dynamic information from the environment in order to ensure successive interaction with it. In the process of evolution, in the mammalian brain have emerged areas with a specific type of functionality that can be regarded as a hierarchical structure processing the visual input. The incoming light is initially converted in the retina into electrical signal by retinal ganglion cells (RGC), passed through the relay station—lateral geniculate nucleus (LGN) and thalamic reticular nucleus (TRN)—to the primary visual cortex (V1) where the visual information splits in two parallel pathways involved in encoding spatial layout and motion (dorsal) and shape (ventral) information. Motion information encoding and interpretation pose serious challenges due to its different sources (self-motion, object motion, or eye movements), the need to integrate local measurements in order to resolve the ambiguities in the incoming dynamic stream of information, but also the need to segregate the signals coming from different objects. The motion information processing is performed predominantly by the middle temporal area (MT) that encodes the speed and direction of the moving objects and the medial superior temporal area (MST) that extracts information about the self-motion of the observer.

Most of the existing motion information processing models are restricted to the interactions between the areas in the dorsal pathway: V1 and MT (e.g., Simoncelli and Heeger, 1998; Bayerl and Neumann, 2004; Bayerl, 2005; Chessa et al., 2016), V1, MT, and MST (Raudies et al., 2012) or MT and MST (Grossberg et al., 1999; Perrone, 2012). Many models consider only the feedforward interactions (e.g., Simoncelli and Heeger, 1998; Solari et al., 2015) disregarding the feedback connectivity; others employ rate-based equations (e.g., Grossberg et al., 2001; Raudies and Neumann, 2010) considering an average number of spikes in a population of neurons.

Here we present spike-timing neural network as an attempt to simulate realistically the interactions between all described processing stages of encoding of dynamic visual information in the human brain. To take into account the process of decision making based on perceived visual information and the preparation of a saccade to the desired location, we included the lateral intraparietal area (LIP) as the output layer. The model behavior was tested with simplified visual stimuli mimicking self-motion with gaze fixed, considering its output as a decision for saccade toward the determined heading direction.

The model is implemented using NEST 2.12.0 simulator (Kunkel et al., 2017).

The paper is organized as follows: Section Model Structure describes briefly the overall model structure; Section Simulation Results reports results from its performance testing; Section Discussion presents a brief discussion of the model limitations and the directions of future work.

## Model Structure

**Figure 1:**
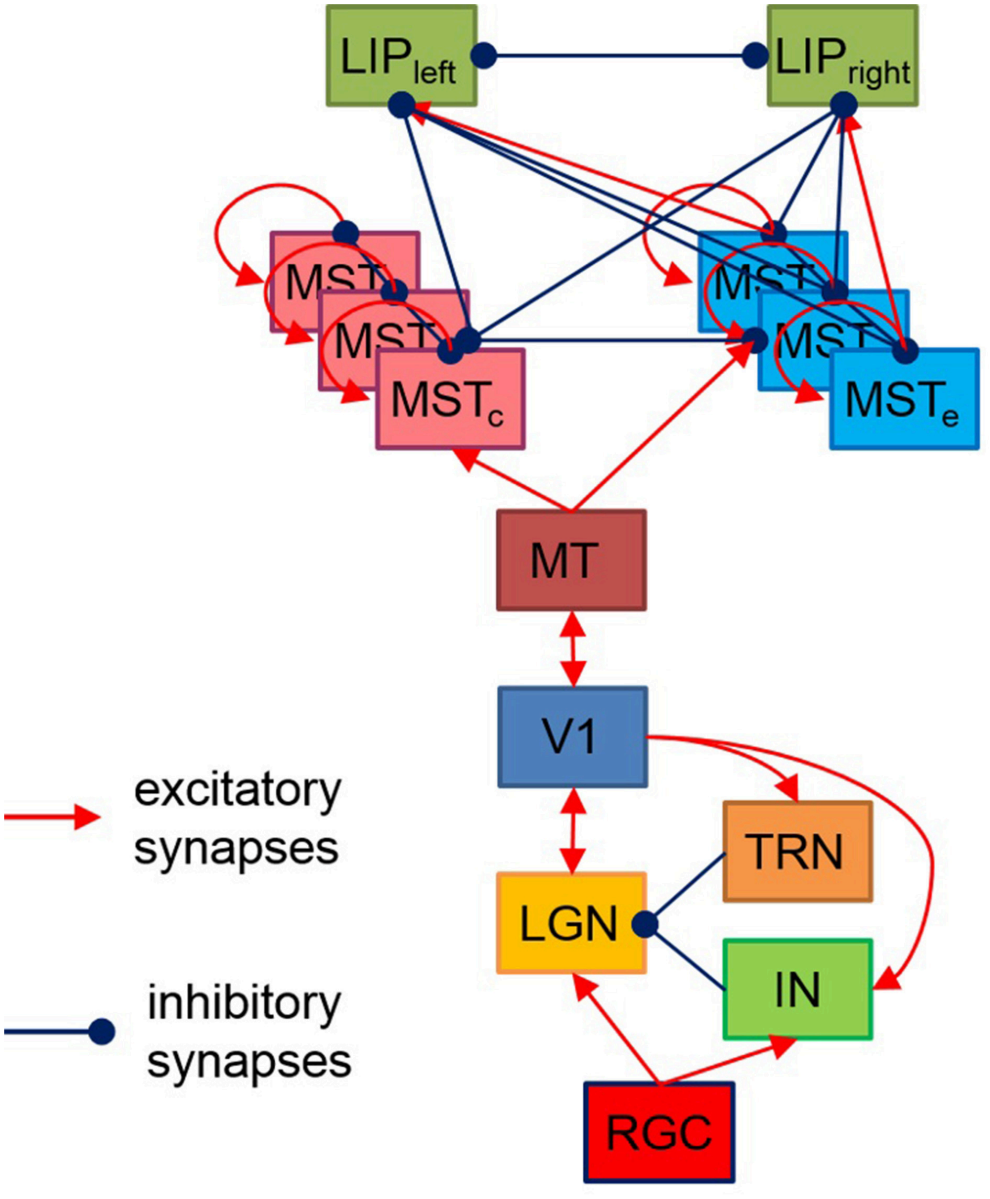
Model structure. Each rectangle denotes a two-dimensional grid of neurons having corresponding to the layer functionality and receptive fields. The model output has two LIP neurons with a preference for preparing a left/right saccade decision based on sensory data. MTSe and MTSc represent MTS neurons with expansion/contraction movement templates.

The proposed here hierarchical model, shown on Figure 1, is based on the available data about brain structures playing a role in visual motion information processing and perceptual decision making, as well as their connectivity. Each layer consists of neurons positioned in a regular two-dimensional grid. The receptive field of each neuron depends both on the function of the layer it belongs to and on its spatial position within its layer.

The reaction of RGC to luminosity changes is simulated by a convolution of a spatiotemporal filter with the images falling on the retina, following models from Troyer et al. (1998) and Kremkow et al. (2016). Its spatial component has a circular shape modeled by a difference of two Gaussians (DOG) while the temporal component has a bi-phasic profile determined by the difference of two Gamma functions. The model contains two layers of ON and OFF RGC and their corresponding LGN and IN/TRN neurons, having identical relative to visual scene positions and opposite [“on-center off-surround” (ON) and “off-center on-surround” (OFF)] receptive fields placed in reverse order like in Kremkow et al. (2016). Each layer consists of totally 400 neurons, positioned on 20 × 20 grid. The continuous current generated by RGC is injected into LGN and IN via one-to-one connections. The structure of direct excitatory synaptic feedforward connectivity between LGN and V1 is also adopted from Kremkow et al. (2016). LGN also receives inhibitory feedback from V1 via IN and TRN according to (Ghodratia et al., 2017).

**Figure 2:**
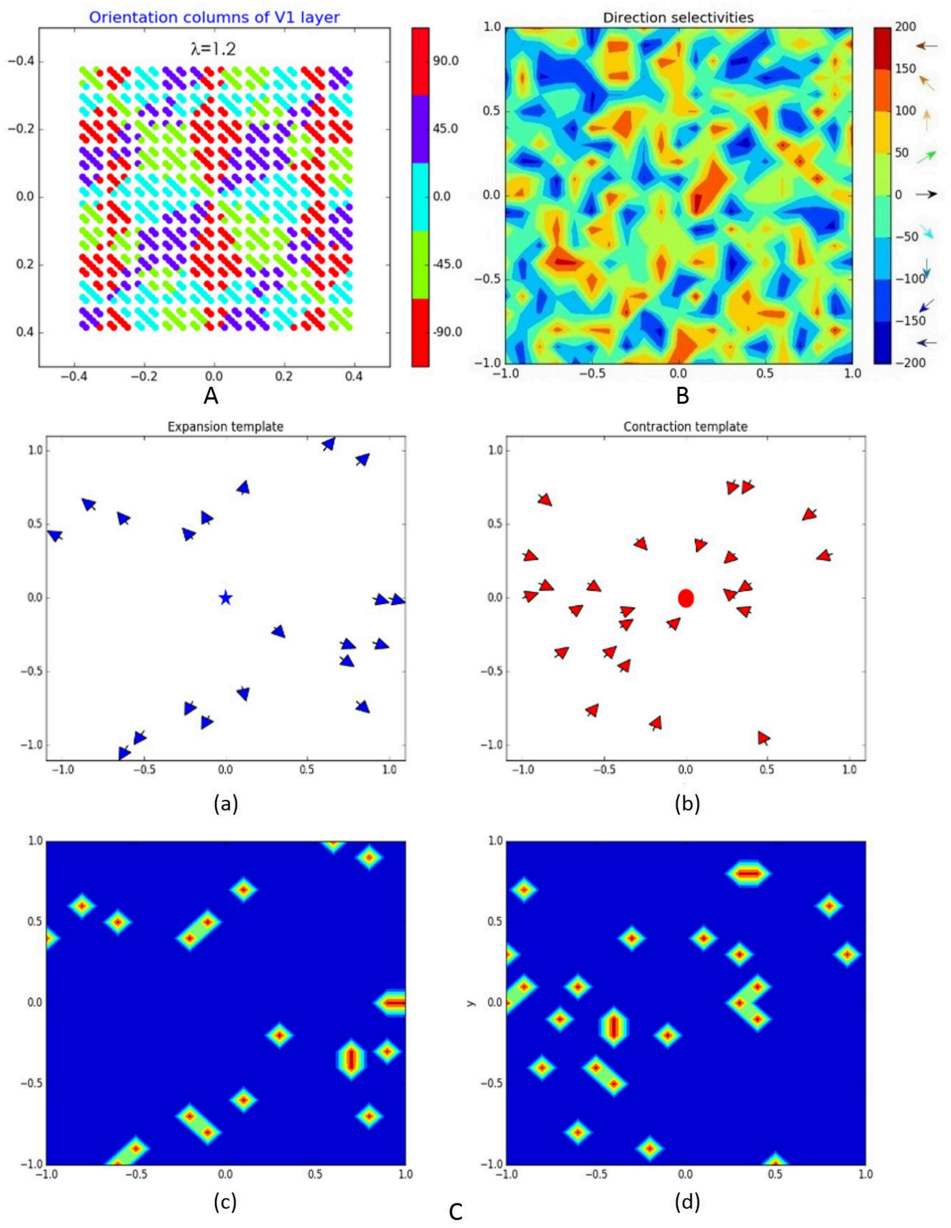
Representation of some of the model layers and their connections: **(A)** orientation columns of V1 layer; **(B)** direction selectivity of MT layer; **(C)** expansion (a) and contraction (b) binary patterns ***T***(**δ**) of MT–MST connections (blue star and red dot denote the expansion/contraction focal points while the arrows show the direction selectivity of MT cells eligible to be connected to corresponding MST pattern) and corresponding to them connection templates ***T***_**e(c)**_ on (c,d).

As in Kremkow et al. (2016), the neurons in V1 are separated into four groups—two exciting and two inhibiting, having a ratio of 4/1 exciting/inhibiting neurons (400/100 in our model) and connected via corresponding excitatory and inhibitory lateral connections. All exciting neurons are positioned at 20 × 20 grid while the 10 × 10 inhibiting neurons are dispersed among them. Being orientation sensitive, V1 neurons have elongated receptive fields defined by Gabor probability function as in Nedelcheva and Koprinkova-Hristova (2019). The “pinwheel structure” of the spatiotemporal maps of the orientations and phases of V1 neurons receptive fields was generated using a relatively new and easily implemented model (Sadeh and Rotter, 2014). An example of V1 orientation map (Nedelcheva and Koprinkova-Hristova, 2019) for a spatial frequency λ of the generating grating stimulus is shown in Figure 2A. Lateral connections in V1 are determined by Gabor correlations between the positions, phases, and orientations of each pair of neurons. As in Kremkow et al. (2016), neurons from inhibitory populations connect preferentially to neurons having a receptive field phase difference of around 180°. In our model, the frequencies, and standard deviations of Gabor filters for lateral connections were chosen so that all neurons in the layer have approximately circular receptive fields.

MT has identical to V1 size and structure and its lateral connections are designed in the same way while the connections from V1 cells depend on the angle ***φ***_ij_ between the orientation preferences of each two cells like in Escobar et al. (2009):


$$\begin{array}{ccc} w_{ij} & = & \{\begin{array}{cc} k_{c}w_{cs}\left(x_{i}^{MT}-x_{j}^{V1},y_{i}^{MT}-y_{j}^{V1}\right)\cos\varphi_{ij}, & 0\leq \varphi_{ij}\leq \frac{\pi }{2}\\ 0, & \frac{\pi }{2}<\varphi_{ij}<\pi \end{array} \end{array}$$

Here **k**_*c*_ is amplification factor and **w**_**cs**_ is weight factor associated with the MT neuron receptive field, modeled as DOG function:


$$\begin{array}{r} w_{\text{cs}}\left(x_{i}^{MT}-x_{j}^{V1},y_{i}^{MT}-y_{j}^{V1}\right)=\frac{a_{c}e^{-\frac{\sqrt{{\left(x_{i}^{MT}-x_{j}^{V1}\right)}^{2}+{\left(y_{i}^{MT}-y_{j}^{V1}\right)}^{2}}}{\sigma_{c}^{2}}}}{\sigma_{c}^{2}}\\ -\frac{a_{s}e^{-\frac{\sqrt{{\left(x_{i}^{MT}-x_{j}^{V1}\right)}^{2}+{\left(y_{i}^{MT}-y_{j}^{V1}\right)}^{2}}}{\sigma_{s}^{2}}}}{\sigma_{s}^{2}} \end{array}$$

where ***a***_***c***_ and ***a***_***s***_ are the center and surround weights and *σ*_***c***_ and *σ*_***s***_ are the corresponding standard deviations. The orientation and phase maps of this layer were generated in the same way as those of V1. An example of direction selectivity map of MT is shown on Figure 2B.

The MST consist of two layers, each one containing 400 neurons positioned on 20 × 20 grid, sensitive to expansion and contraction movement patterns, respectively, like in Layton and Fajen (2017). Each MST cell has assigned an expansion/contraction connection template ***T***_***e*(*c*)**_ having a circular shape with width **d** and focal point (***x***_***e*(*c*)**_**,*y***_***e*(*c*)**_) at MT as follows:


$$\begin{array}{c} T_{e(c)}\left(x_{e(c)},y_{e(c)},x_{MT},y_{MT}\right)=T\left(\delta \right)e^{-d\left({\left(x_{e(c)}-x_{MT}\right)}^{2}+{\left(y_{e(c)}-y_{MT}\right)}^{2}\right)}\\ \delta =arctg\frac{y_{e(c)}-y_{MT}}{x_{e(c)}-x_{MT}} \end{array}$$

Here *δ* is the radial template angle determined by the position of each MT cell (**x**_***MT***_**,*y***_***MT***_) and the given pattern expansion/contraction focal point. The binary pattern variable ***T***(*δ*) is non-zero only if the corresponding MT cell has direction preference toward/against the contraction/expansion center of MST. Figure 2C shows examples of MT cells (with direction selectivity presented by arrows at corresponding positions) that are eligible for connection to corresponding expansion/contraction MST cells having focal points marked by blue star and red dot [(a) and (b)] and the corresponding connection templates [(c) and (d)].

The MST neurons have on-center receptive fields with standard deviation **σ**. Each MST neuron collects inputs from MT cells corresponding to its pattern template as follows:


$$\begin{array}{r} w_{e(c)}\left(x_{MT},y_{MT},x_{MST},y_{MST}\right)=T_{e(c)}\left(x_{e(c)},y_{e(c)},x_{MT},y_{MT}\right)\\ \frac{e^{-\frac{{\left(x_{MT}-x_{MST}\right)}^{2}+{\left(y_{MT}-y_{MST}\right)}^{2}}{2\sigma_{I}^{2}}}}{\sqrt{2\pi \sigma_{I}^{2}}} \end{array}$$

Both layers have intra- and interlayer excitatory/inhibitory recurrent connections between cells having similar/different sensitivity as shown on Figure 1.

These lateral connections are determined based on neurons' positions and template similarities. All neurons have Gaussian receptive fields. Connections within expansion/contraction layers are excitatory or inhibitory in dependence on their focal points similarity as follows:


$$\begin{array}{l} w_{e(c)}^{intra}\left(x_{MST}^{1},y_{MST}^{1},x_{MST}^{2},y_{MST}^{2}\right)\\ =\{\begin{array}{cc} +e^{-\frac{{\left(x_{MST}^{1}-x_{MST}^{2}\right)}^{2}+{\left(y_{MST}^{1}-xy_{MST}^{2}\right)}^{2}}{2\sigma_{ts}^{2}}}, & if\;x_{e(c)}^{1}=x_{e(c)}^{2}and\;y_{e(c)}^{1}=y_{e(c)}^{2}\\ -e^{-\frac{{\left(x_{MST}^{1}-x_{MST}^{2}\right)}^{2}+{\left(y_{MST}^{1}-xy_{MST}^{2}\right)}^{2}}{2\sigma_{ts}^{2}}}, & otherwise \end{array} \end{array}$$

Connections between expansion and contraction layers are all inhibitory and depend both on similarities of their positions and focal points as follows:


$$\begin{array}{l} w_{e(c)}^{c(e)}\left(x_{MST}^{e},y_{MST}^{e},x_{MST}^{c},y_{MST}^{c}\right)=\\ -e^{-\frac{{\left(x_{MST}^{e}-x_{MST}^{c}\right)}^{2}+{\left(y_{MST}^{e}-xy_{MST}^{c}\right)}^{2}}{2\sigma_{ts}^{2}}}e^{-\frac{{\left(x_{e(c)}^{e}-x_{e(c)}^{c}\right)}^{2}+{\left(y_{e(c)}^{e}-xy_{e(c)}^{c}\right)}^{2}}{2\sigma_{ts}^{2}}} \end{array}$$

In present work, we used only three focal points having identical vertical positions ***y***_***e*(c)**_ = **0**.

Since our model aims to decide whether the expansion center of a moving dot stimulus is left or right from the stimulus center, here we proposed a task-dependent design of excitatory/inhibitory connections from MST expansion/contraction layers to the two LIP sub-regions whose increased firing rate corresponds to two taken decisions for two alternative motor responses—eye movement to the left or to the right. Both LIP areas are modeled by two neurons receiving excitatory input from MST expansion layer neurons having focal points corresponding to their decision responses (left or right) and inhibitory input from all other MST neurons. There are also lateral inhibitory connections between both LIP areas (Figure 1).

For the neurons in LGN conductance-based leaky integrate-and-fire neuron model as in Casti et al. (2008) (iaf_chxk_2008 in NEST) was adopted. For the rest of neurons, leaky integrate-and-fire model with exponential shaped postsynaptic currents according to Tsodyks et al. (2000) (iaf_psc_exp in NEST) was used. All connection parameters are the same as in the cited literature sources.

## Simulation Results

In our previous work (Nedelcheva and Koprinkova-Hristova, 2019) we tested orientation selectivity of V1 in order to tune parameters of receptive fields of both LGN and V1 and the spatial frequency of V1 orientation columns using moving bar stimuli with two orientations. In Koprinkova-Hristova et al. (2018) we demonstrated that feedback inhibitory connections from V1 to LGN via TRN/IN modulates V1 neurons selectivity.

**Figure 3:**
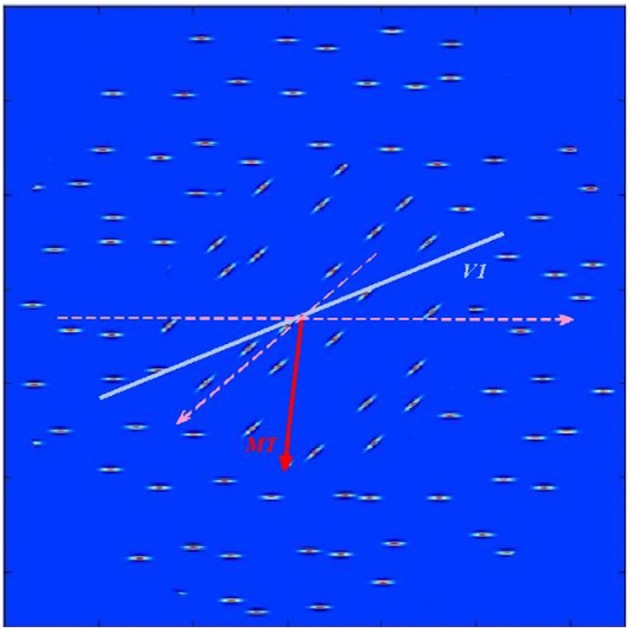
Test stimulus consisting of horizontal and diagonal bars moving parallel to the bar orientations in each of the two stimulus regions as shown by dashed pink lines). The blue thick line shows estimated in V1 layer average orientation of the stimulus. The red arrow points toward estimated in MT layer average direction of bar movement within the stimulus.

Further, we tested responses of MT using a stimulus composed of horizontal and diagonal bars moving with equal speed along different directions. To evaluate model responses, the vector-averaged population decoding of V1, and MT was determined as in (Webb et al., 2010):


$$\begin{array}{ccccc} O & R_{est} & = & arctg & \frac{\sum_{i}n_{i}\sin\theta_{i}}{\sum_{i}n_{i}\cos\theta_{i}} \end{array}$$

where **n**_**i**_ is the total number of spikes generated by neurons having sensitivity to i-th orientation/direction. Estimated orientation and direction of stimulus shown on Figure 3 in V1 and MT were 50.83° and 93.26° and correspond approximately to the mean values of the underlying stimulus characteristics.

The overall model was tested using visual stimulation simulating an observer's motion on a linear trajectory with eyes fixed in the heading direction. The stimuli consisted of 50 moving dots (36 of which moved radially and 14 with random movement directions) having expansion centers left or right from the visual scene center. Each dot lasted for 100 ms after which it was re-positioned randomly preserving its motion direction. On every frame, only one-third of the dots changed position. Variations of stimuli having seven expansion center positions ranging from 0.67 to 4.67° of arc (20–140 pixels) to the left or to the right of the screen center were generated. A detailed description of the experiment and the results with human subjects are given in Bocheva et al. (2018).

**Figure 4:**
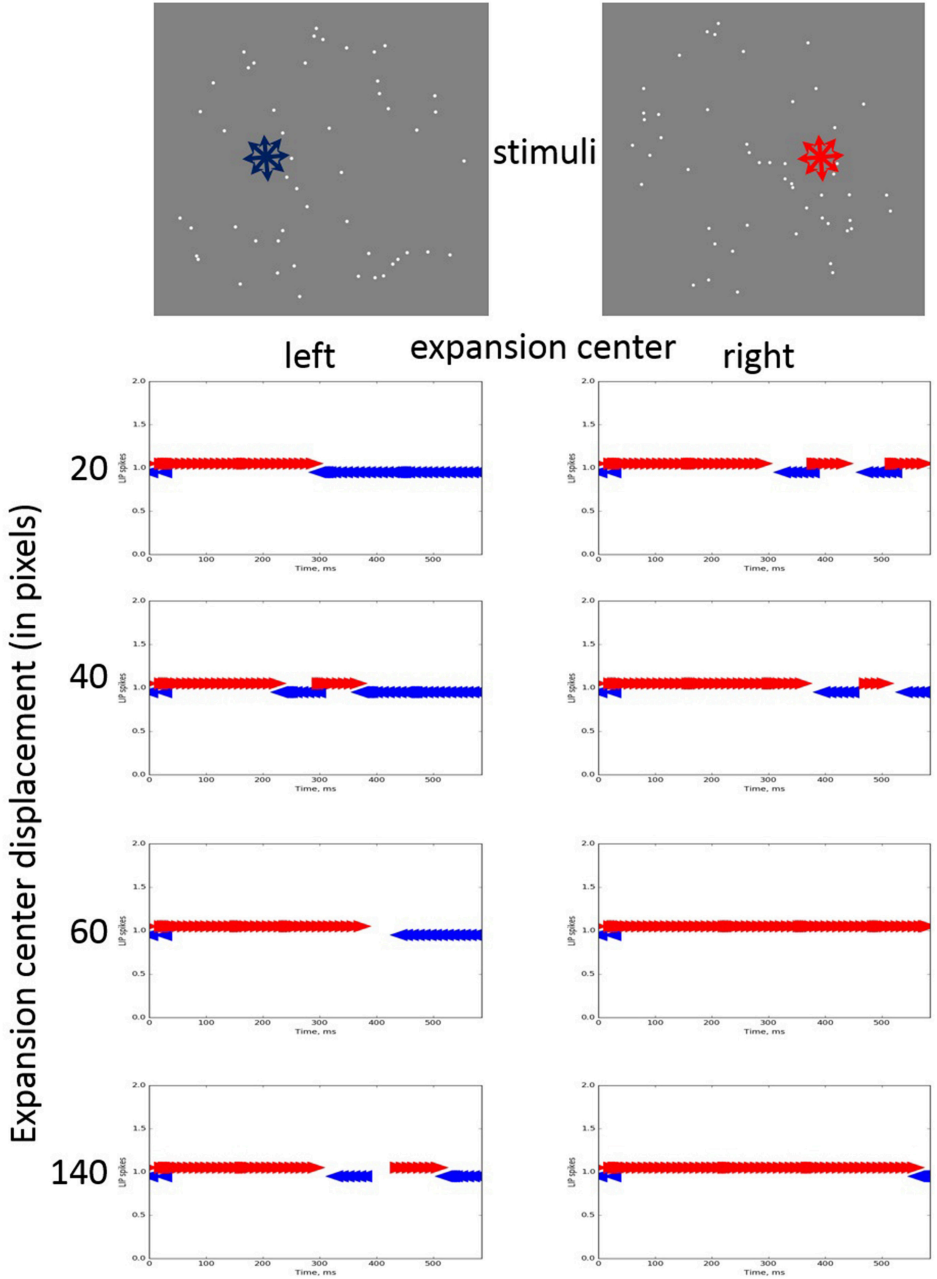
LIP neurons spikes induced by moving dot stimuli (on the top) having expansion centers (denoted by stars indicating the predominant directions of motion of the dots) with varying displacements to the left or to the right from the screen center. The arrows pointing to the left and right denote the corresponding neuron spikes (blue for the left and red for the right, respectively).

Spike trains generated by both LIP neurons (left and right) in response to the stimuli with varying center displacements (in pixels) moving for a duration of 600 ms are presented on Figure 4.

The simulation data showed that in all cases after a period of uncertainty the firing rate in the LIP area corresponding to the correct expansion center position is higher. The moment when correct decision starts to prevail depends on the task difficulty, i.e., the displacement magnitude. The LIP neuron reaching the correct decision has a shorter period of uncertainty with length inversely proportional to the center displacement magnitude. We also observed asymmetrical behavior of left/right LIP areas: the right decision is taken faster while for the left the model needed 300–400 ms to switch to the correct decision for intermediate displacements and longer time for the largest one.

## Discussion

The model has several limitations. We have focused only on the dorsal pathway and disregarded the interactions between the two visual pathways. However, the stimulation we used for model testing does not require additional complication even though its performance might be better at the MT stage if the information about the motion boundaries between the two regions of the stimulus configuration were extracted and supplied by the ventral pathway. The model parameters are based predominately on the data published in the literature. They have to be additionally tuned to represent the human performance in behavioral experiments with the same type of stimuli, as those reported by Bocheva et al. (2018).

The simulation data were obtained for fixed stimulus duration and suggest that the correct choice is achieved in <600 ms. However, the human observers, especially the older ones, needed more time to make a response. Only about 10 percent of the responses were shorter than 600 ms and only 53.4% of these short responses were correct. While this suggests that the model outperforms the observers in accuracy and speed and is more effective in integrating the spatial and temporal information than the human observers, it needs to be emphasized that the reaction time of the human observers contains also non-decision components that involve the preparation of the motor response. Indeed, our data show that the component of the reaction time not related to decision-making is on average 342 ms for the young age group, 520 ms for the middle aged and 825 ms for the elderly. This non-decision time could not be taken into account in the model as it simulates only the decision making based on the accumulation of sensory information. In the future, we will test the model for longer stimulus duration and implement an ability to make a choice after the stimulus extinction.

In spite of its limitations, our model reproduced certain characteristics of the behavioral data like the trend for increased response times with the decrease in expansion center displacement.

We need to emphasize also that more elaborated stimuli were used for model testing than the typically used gratings or random dot patterns with the supposition that if the model performs well with these stimuli, it will perform well with simpler stimuli as well. However, even though our stimuli are more complex than the typical ones, they are simplified versions of the stimulation experienced in natural conditions and tasks. Additional tests with a larger set of stimuli are needed in order to improve model behavior. This will allow adjusting model parameters so that they replicate the age differences in performance in different tasks in dynamic conditions. The involvement of other brain structures contributing to saccade programming is another direction in our future work.

## Author Contributions

PK-H and NB contributed conception and design of the study. NB and MS developed visual stimuli. PK-H and SN performed the programming of the model in NEST. NB wrote the introduction, stimulus description, and discussion sections of the manuscript. PK-H wrote the model description sections. All authors contributed to manuscript revision, read and approved the submitted version.

### Conflict of Interest Statement

The authors declare that the research was conducted in the absence of any commercial or financial relationships that could be construed as a potential conflict of interest.
